# Polyphenolic Composition, Antioxidant Activity, and Cytotoxic Effect of Male Floral Buds from Three *Populus* Species Growing in the South of Romania

**DOI:** 10.3390/molecules30040913

**Published:** 2025-02-16

**Authors:** Mona Luciana Gălăţanu, Mariana Panţuroiu, Luiza Mădălina Cima, Ana Maria Neculai, Emilia Pănuş, Coralia Bleotu, Cristian Mihai Enescu, Ion Mircioiu, Roxana Măriuca Gavriloaia, Sorina Nicoleta Aurică, Mirela Claudia Rîmbu, Roxana Colette Sandulovici

**Affiliations:** 1 Faculty of Pharmacy, Titu Maiorescu University, Sincai Boulevard, No. 16, 040314 Bucharest, Romania; luciana.galatanu@prof.utm.ro (M.L.G.); luiza.cima@prof.utm.ro (L.M.C.); ion.mircioiu@prof.utm.ro (I.M.); roxana.gavriloaia@prof.utm.ro (R.M.G.); sorina.aurica@gmail.com (S.N.A.); mirela.rimbu@prof.utm.ro (M.C.R.); roxana.sandulovici@prof.utm.ro (R.C.S.); 2 Department of Biochemistry, Faculty of Medicine, Ovidius University of Constanta, Universitatii Street, No. 1, 900470 Constanta, Romania; anamneculai89@gmail.com (A.M.N.); temilia2@yahoo.com (E.P.); 3 Microbiology and Molecular Biology Laboratory, Public Health Constanta, 900587 Constanța, Romania; 4 Cellular and Molecular Pathology Department, Stefan S. Nicolau Institute of Virology, Romanian Academy, 030304 Bucharest, Romania; cbleotu@yahoo.com; 5 The Research Institute, University of Bucharest, 030018 Bucharest, Romania; 6 Department of Life, Medical and Agricultural Sciences, Biological Sciences Section, Academy of Romanian Scientists, 050044 Bucharest, Romania; 7 Department of Soil Sciences, University of Agronomic Sciences and Veterinary Medicine of Bucharest, 59 Mărăști Boulevard, 011464 Bucharest, Romania; mihai.enescu@agro-bucuresti.ro

**Keywords:** *Populus*, polyphenols, flavonoids, antioxidant capacity, cytotoxic effect

## Abstract

Three poplar species widely distributed in southern Romania were investigated for their chemical composition and bioactivity. Male buds from black poplar (*Populus nigra* L.), white poplar (*Populus alba* L.), and Euroamerican hybrid poplar (*Populus* × *euramericana* (Dode) Guinier.) were analyzed using HPTLC, HPLC, GC-MS, and spectrophotometric assays. The analysis revealed predominantly polyphenolic compounds, including phenolic acids and flavonoids, secondary metabolites recognized for their antioxidant properties, particularly valuable in alleviating oxidative stress disorders. Heavy metal content was measured using atomic absorption spectroscopy, and antioxidant capacity was assessed through DPPH and FRAP assays alongside a cytotoxicity evaluation. Polyphenolic content ranged from 19.26 to 33.37 mg GAE/g DW and flavonoid content from 2.15 to 4.45 mg RE/g DW. All three species demonstrated notable antioxidant capacity and cytotoxic activity. Hydroethanolic extracts of *P. nigra* and *P. euramericana* showed higher antioxidant activity than aqueous extracts, with *P. nigra* achieving the lowest IC_50_ value overall, highlighting the influence of solvent choice on antioxidant efficacy. Furthermore, poplar hydroethanolic extracts exhibited concentration-dependent cytotoxicity against fibroblast-like human osteosarcoma MG63 cell lines, with IC_50_ values of 42.55 µg/mL for *P. nigra*, 40.87 µg/mL for *P.* × *euramericana*, and 132.49 µg/mL for *P. alba*, underscoring significant interspecies variability in cytotoxic potency. These findings suggest that male floral buds from Romanian poplar species may serve as valuable sources of bioactive compounds with therapeutic potential.

## 1. Introduction

The genus *Populus* belongs to the Salicaceae family in the *Angiosperms* clade. It comprises 22–45 taxa, although taxonomists have no agreement regarding the exact number [1]. Poplars are deciduous trees growing mostly in the northern zones of the Earth, along watercourses, in meadows, and in urban areas on plains and low hills under 1600 m of altitude [2]. Among the *Populus* species, the black poplar (*Populus nigra* L.), white poplar (*Populus alba* L.), and Euroamerican hybrid poplar (*Populus* × *euramericana*) cover large areas across Europe [3,4].

*Populus* species have columnar crowns and simple leaves with different shapes (triangular mostly). The unisexual flowers, grouped in long, pedunculated catkins without calyces or corollas, appear in early spring from buds before the leaves. The fruits are dehiscent capsules bearing numerous long, white, hairy seeds. The pollination and spreading of the seeds are achieved by the wind [5,6,7,8]. There are a few differences between poplar species, including the aspect of the leaves (color, dimensions, pubescence), bark, and flower spikes.

The buds and the bark of poplar have been used in folk medicine for many years due to their powerful active biocompounds.

Many studies have been conducted to discover the black poplar bud’s chemical composition. These studies have demonstrated high levels of phenolic acids such as caffeic acid, ferulic acid, *p*-coumaric acid, cinnamic acid, chicoric acid, ellagic acid, rosmarinic acid, and their derivates and an increased amount of flavonoids such as flavanones (pinocembrin, pinostrobin, pinobanksin, naringenin), flavones (apigenin, chrysin, tectochrysin, luteolin), flavonols (kaempferol, quercetin, galangin), flavanonols (taxifolin), and glycosides (myrcitrin, luteolin7-*O*-glucoside) [9,10,11,12,13,14,15]. The essential oil obtained from black poplar buds from Croatia and Spain mainly comprises sesquiterpenoids (α- and β-eudesmol, γ-selinene, δ- and γ-cadinene, α-elemene, curcumene, bisabolene, farnesol, humulene) and a few monoterpenoids (1,8-cineole) [16,17]. Phenolic glycosides such as salicin, salireposide, and benzoylsalicin (populin) are essential compounds of black poplar buds and other Salicaceae family members. Salicin can be hydrolyzed to D-glucose, and saligenin (salicyl alcohol) and has significant anti-rheumatic and analgesic effects [18]. Other biocompounds such as tannins, saponins, resins, vanillin, syringaldehyde, acetophenone, and alkaloids can be found in black poplar buds [15,19,20]. The resinous exudate of black poplar buds represents one of the production bases of European propolis [21,22].

The bark of black poplar has essential amounts of lignin of the *p*—hydroxybenzoate type, as well as other lignins, salicin, flavonoids, polyphenols, hydrolysable tannins, phenolic monomers (catechol vanillin), sugars, alkanes, alcohols, and carboxylic acids [23,24,25].

The phytochemical profile of white and Euroamerican hybrid poplars has not been rigorously studied. It is known that white poplar bark contains flavonoids, phenolic acids, salicin derivatives, tannins, and polysaccharides [26], while male catkins contain large amounts of anthocyanins (cyanidin 3-O-glucoside) [27]. Tawfeek et al. identified in the leaves and young shoots of white poplar the presence of polyphenolic derivates (protocatechuic acid, trans *p*-coumaric acid methyl ester), flavonoids and their glycosides (naringenin, aromadendrin, kaempferol, quercetin, isorhamnetin 3-*O*-β-d-rutinoside), phenolic glycosides and derivates (salicin, salicyl ether, 2-*O*-acetyl salicin, tremuloidin), and other compounds (trans-1,2 cyclohexanediol, n-nonadecanol-1, grandidentatin) [28]. The buds of Euroamerican poplar contain flavonoids and polyphenolic acids [29], while the leaves produce and release volatile terpenes such as β-ocimene and linalool [30].

Black poplar has many traditional uses due to its analgesic–antipyretic, anti-inflammatory, diuretic, astringent, and cicatrizing action, and recent clinical studies have demonstrated new important pharmacological effects [6,10,14].

*Populus nigra* extract was tested in vitro on lung cancer A 549, NCI-H 292, and breast cancer MCF-7 cell lines, which indicated its anticancer activity [31,32]. Other studies have highlighted antitumor effect of various *Populus* species: *P. yunnanensis* on osteosarcoma lines [33] and *P. euphratica* on oral cancer lines [34].

The anti-inflammatory action of black poplar bud extract is based on its numerous phenolic compounds, and it has been observed in human gingival fibroblasts, with a mechanism of decreasing the production of pro-inflammatory cytokines IL-6 and IL-1β [35]. This can be correlated with the strong antioxidant capacity of black poplar buds demonstrated in vitro [17,19].

Also, black poplar buds show complex antimicrobial action, namely antibacterial effects on different strains of bacteria—*Staphylococcus aureus*, *Streptococcus pyogenes*, *Enterococcus faecalis*, *Listeria monocytogenes*, and *Bacillus subtills*—and on fungi—*Candida, Aspergillus*, and *Fusarium* [31].

Other recently discovered effects of black poplar buds include antidiabetic [36], hepatoprotective, vasorelaxant, and hypouricemic action [36,37].

White poplar has been used in traditional medicine as an oral decoction or ointment for depurative, anti-inflammatory, and wound-healing action in rheumatic pains, skin lesions, hemorrhoids, and herpes [28].

*P. alba* leaves and young shoots show pharmacological activities such as antioxidant capacity, cytotoxicity, and weak antibacterial effects [28,38].

Euroamerican poplar buds show activity against Gram-positive bacteria and fungi, as Okińczyc et al. revealed in their in vitro studies [29].

Modern biology’s major challenge is identifying how environmental stress affects plant organisms [39,40,41,42]. With the increasing environmental pollution caused by heavy metals, there has been a significant increase in interest in studying their effects on plant adaptability and tolerance [43]. Industrial activities such as mining, fuel combustion, and by-product contamination are among the primary sources of heavy metal pollution in the environment. The European Union has identified certain heavy metals of particular concern, with extensive research conducted on metals such as Cd. However, there is still a lack of knowledge on the phytotoxicity of other metals such as Hg, including their mechanisms and overall impact [44]. Lead, along with cadmium, are heavy metals that are not necessary for the functioning of plant organisms and can also be harmful [45,46]. Before extraction, it is crucial to determine the heavy metal contents of plant samples, especially when considering the renowned capability of poplar to remove heavy metals from soil and water [15].

The aim of this study is to realize a complex chemical analysis of the male floral buds from the species of poplar growing abundantly in the South of Romania, including the identification of the main biocompounds, the analysis of flavonoids and polyphenols, the detection of heavy metals, and the assessment of biological activities.

## 2. Results

This study involved the preparation of two types of extracts—hydroethanolic and aqueous—from the male buds of poplar. Preliminary spectrophotometric analyses quantified key bioactive compounds, including flavonoids and total polyphenols, alongside antioxidant activity, to identify the most relevant extract for the study’s objectives. Based on the results, hydroethanolic extracts from *P. nigra*, *P. × euramericana*, and *P. alba* were selected for further analysis. Advanced analytical techniques, including HPTLC, HPLC, and GC-MS, were used for their characterization. Additionally, the heavy metal content and cytotoxic effects of the extracts were evaluated.

### 2.1. Determination of Total Flavonoid Content (TFC) and Total Polyphenol Content (TPC) Using Spectrophotometric Methods

The total polyphenolic and flavonoid contents in the male flower buds of the three analyzed poplar species were determined and are presented in Table 1. The values are expressed in milligrams of rutin equivalent per gram of dried plant material (mg RE/g DW). The flavonoid content varied depending on the *Populus* species and the extraction solvent. For extracts obtained by refluxing with 50% hydroethanolic solvent (50% HE), *P. nigra* showed the highest flavonoid content at 4.45 mg/g, followed by *P. × euramericana* with 3.93 mg/g and *P. alba* with 2.94 mg/g. The flavonoid content was generally lower for all species in the extracts obtained by water refluxing. *P. nigra* had a content of 2.79 mg/g, while *P. × euramericana* had 3.07 mg/g, and *P. alba* had the lowest flavone content at 2.15 mg/g. These results suggest that 50% HE is more effective for flavonoid extraction in all three species studied.

The total polyphenol content, expressed in mg gallic acid equivalent per gram dried plant material (mg GAE/g DW), also varied by *Populus* species and extraction solvent. Gallic and caffeic acids were used as standards to express the total phenolic content, providing a broader and more comprehensive assessment of the polyphenol composition in the samples. Their selection accounts for the structural diversity of phenolic compounds, ensuring a more accurate representation of the overall phenolic profile.

For extracts obtained with 50% hydroethanolic solvent (50% HE) by refluxing*, P. × euramericana* showed the highest polyphenol content (33.37 mg/g), followed closely by *P. nigra* (32.83 mg/g), while *P. alba* showed the lowest content (20.92 mg/g). In water-refluxed extracts, the total polyphenol content was generally lower: *P. × euramericana* had the highest value (27.90 mg/g), followed by *P. nigra* (23.83 mg/g) and *P. alba* with the lowest content (19.26 mg/g). When the total polyphenol content was expressed in caffeic acid equivalents (mg CAE/g DW) for the 50% hydroethanolic extracts, *P. nigra* showed the highest content (32.91 mg/g), followed by *P. × euramericana* (27.90 mg/g) and *P. alba* (19.26 mg/g). In water-refluxed extracts, the total polyphenol content, expressed in caffeic acid equivalents, was also highest in *P. × euramericana* (30.08 mg/g), followed by *P. nigra* (21.99 mg/g) and *P. alba* (19.62 mg/g). 

These findings highlight how extraction methods and regional factors influence the phytochemical profiles of extracts derived from poplar species [11,29].

### 2.2. Antioxidant Capacity

#### 2.2.1. DPPH-Scavenging Capacity

The free radical-scavenging activity of the three poplar species was assessed using the DPPH assay. The IC_50_ values were determined for both aqueous extracts and 50% hydroethanolic extracts. In 50% hydroethanolic extracts, *P. nigra* exhibited the highest antioxidant activity (33.440 µg/mL), closely followed by *P. × euramericana*, which had a slightly higher IC_50_ value (33.900 µg/mL) but still demonstrated strong antioxidant capacity. In contrast, *P. alba* had the highest IC_50_ value (51.250 µg/mL), indicating comparatively lower antioxidant activity in this solvent system. Aqueous extracts followed a similar trend: *P. nigra* and *P. × euramericana* displayed similar antioxidant activities (47.667 µg/mL and 48.355 µg/mL, respectively), while *P. alba* again showed the lowest activity (59.000 µg/mL). For all species, IC_50_ values were higher in aqueous extracts than in 50% hydroethanolic extracts. This difference was most pronounced for *P. nigra* and *P. × euramericana,* where the IC_50_ values in 50% HE were about 30% lower than those in water. In contrast, *P. alba* exhibited a smaller reduction in IC_50_ between the two solvent systems, suggesting that solvent choice had less impact on its antioxidant potential.

#### 2.2.2. FRAP Antioxidant Capacity

The antioxidant activity of the three poplar species was also evaluated using the FRAP method, with variations observed both by species and by the solvent used for extraction. For the extracts obtained using water as a solvent, *P. nigra* exhibited an antioxidant activity of 1.708 mmol Fe^2+^/g dry weight (DW), *P.* × *euramericana* exhibited 1.836 mmol Fe^2+^/g DW, and *P. alba* demonstrated 0.624 mmol Fe^2+^/g DW. In contrast, extracts obtained using 50% hydroethanolic solvent showed higher antioxidant capacities in all species: *P. nigra* exhibited 2.394 mmol Fe^2+^/g DW, *P.* × *euramericana* demonstrated 2.066 mmol Fe^2+^/g DW, and *P. alba* exhibited 1.101 mmol Fe^2+^/g DW. These results are in accordance with the previous determinations (TPC, TFC, DPPH).

### 2.3. Chemical Analysis of the Main Components

#### 2.3.1. HPTLC Analysis of 50% Hydroethanolic Extracts

**Figure 1:**
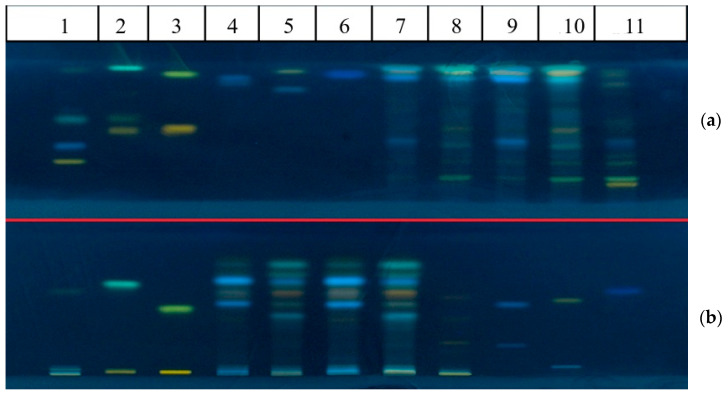
TLC profile of hydroethanolic extracts from poplar buds. (**a**) (Silicagel 60 F 254, ethyl acetate/formic acid/acetic acid/water, 100:11:11:26, *v*/*v*/*v*/*v*) standard mixtures: 1—rutin, chlorogenic acid, apigenin-7-glucoside, apigenin; 2—isoquercetin, kaempferol-3-glucoside, kaempferol; 3—luteolin-7-glucoside, luteolin; 4—caffeic acid, rosmarinic acid; 5—isochlorogenic acid, quercetin; 6—ferulic acid, p-coumaric acid. Extracts: 7—*P. nigra* extract (2 µL); 8—*P. × eurameticana* extract (2 µL); 9—*P. nigra* extract (4 µL); 10—*P. × euramericana* extract (4 µL); 11—*P. alba* extract (4 µL). (**b**) (Silicagel 60 F 254, hexane/ethyl acetate/formic acid, 18:20:2, *v*/*v*/*v*) standard mixtures: 1—rutin, chlorogenic acid, apigenin-7-glucoside, apigenin; 2—isoquercetin, kaempfetol-3-glucoside, kaempferol; 3—luteolin-7-glucoside, luteolin; 9—caffeic acid, rosmarinic acid; 10—isochlorogenic acid, quercetin; 11—ferulic acid, p-coumaric acid. Extracts: 4—*P. nigra* extract (2 µL); 5—*P. × euramericana* extract (2 µL); 6—*P. nigra* extract (4 µL); 7—*P. × euramericana* extract (4 µL); 8—*P. alba* extract (4 μL).

The polyphenolic composition of the flower buds from three *Populus* species was analyzed and compared using an optimized thin-layer chromatography (TLC) method. Preliminary separations were performed by TLC and high-performance thin-layer chromatography (HPTLC) using standard mixtures containing flavones (apigenin, apigenin-7-glucoside, luteolin, luteolin-7-glucoside), flavonols (kaempferol, kaempferol-3-glucoside, isoquercetin, rutin), and phenolic acids (caffeic acid, chlorogenic acid, rosmarinic acid, isochlorogenic acid, *p*-coumaric acid). Two mobile phase systems were used: mobile phase 1 (ethyl acetate/formic acid/acetic acid/water, 100:11:11:26, *v*/*v*/*v*/*v*) and mobile phase 2 (hexane/ethyl acetate/formic acid, 18:20:2, *v*/*v*/*v*) (Figure 1). Table 2 shows the reference substances and their retention factors (R_f_) to facilitate the accurate identification and comparison of flavonoids and polyphenolic compounds in *Populus* species.

The HPTLC-based method allowed for the identification of different flavonoids and phenolic acids in flower buds of the *Populus* species studied. In *P. nigra*, the identified compounds included chlorogenic acid, apigenin, quercetin, caffeic acid, and kaempferol. In *P. × euramericana*, the detected compounds were apigenin, isoquercetin, luteolin, kaempferol, chlorogenic acid, and isochlorogenic acid. For *P. alba*, rutin, chlorogenic acid, apigenin, and quercetin were identified. Correlating retention factors with standard compounds achieved the precise identification of flavonoid and phenolic acid polyphenolic profiles.

#### 2.3.2. HPLC-DAD Analysis of 50% Hydroethanolic Extracts

**Figure 2:**
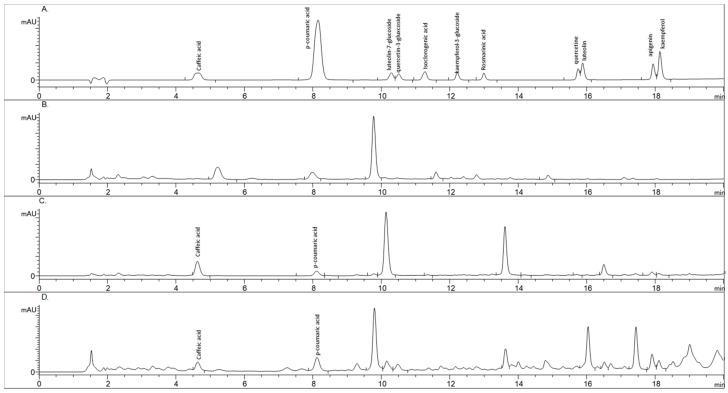
HPLC-DAD chromatograms recorded at 305 nm wavelength: (**A**) standard polyphenolic compound mix; (**B**) hydroethanolic extract of white poplar; (**C**) hydroethanolic extract of black poplar; and (**D**) hydroethanolic extract of Euroamerican poplar.

This method provides a robust approach for analyzing the complex mixture of phenolic acids and flavonoids in *Populus* sp. extracts. Using an elution gradient and simultaneous wavelength monitoring ensures the efficient separation and accurate detection of bioactive compounds. The image below (Figure 2) shows the chromatographic fingerprints of the extracts studied. Table 3 provides a detailed overview of the retention times of reference compounds identified through HPLC analysis. The HPLC-DAD chromatogram profile of the mixed reference solution can be found as reference material. For compounds in samples with retention times differing from those of the reference substances but showing spectral similarity to the reference compounds, it can be inferred by extrapolation that they may be derivates of identified compounds. These chromatograms are presented in the Supplementary Material.

The HPLC-DAD analysis using the external standard method [15,29] identified and quantified caffeic acid and *p*-coumaric acid in the extracts of three *Populus* species. Using the HPLC method, the concentration of caffeic acid in black poplar extract was determined to be 0.56 mg/mL. In contrast, the Euroamerican poplar extract contained a significantly lower concentration of 0.06 mg/mL, nearly ten times lower. The *p*-coumaric acid concentration in black poplar extract was 0.054 mg/mL, compared to 0.025 mg/mL in the Euroamerican poplar extract, showing that black poplar contains roughly double the amount of *p*-coumaric acid. These differences in the concentrations of caffeic and *p*-coumaric acids between the two poplar species emphasize their distinct chemical diversity and bioactive potential.

Neither caffeic acid nor *p*-coumaric acid was detected in the white poplar extract.

#### 2.3.3. GC-MS Analysis of 50% Hydroethanolic Extracts

The 50% hydroethanolic extracts from the buds of the three poplar species contained a range of compounds, as detailed in Table 4. The chromatograms highlight the retention times of silylated derivatives, including phenolic acids, flavonoids, sugars, and organic acids. These peaks represent TMS (trimethylsilyl) derivatives, enhancing the volatility and detectability of the metabolites in GC-MS analysis. The chromatograms are provided as figures in the Supplementary Material.

### 2.4. Heavy Metal Detection

#### 2.4.1. Mercury Analysis

Table 5 shows the amounts of mercury found in the male floral buds of the three species of poplar.

The concentration of mercury varies slightly among the three poplar samples. White poplar shows the highest mean mercury concentration (0.0082 mg/kg), followed by Euromerican poplar (0.0066 mg/kg) and black poplar (0.0055 mg/kg). These differences could be attributed to species-specific physiological traits such as root structure, uptake mechanisms, and detoxification processes. White poplar, being a fast-growing and adaptable species, may have a higher capacity to absorb and tolerate mercury, explaining its slightly higher concentration.

#### 2.4.2. Heavy Metal Analysis

The concentrations of heavy metals found in the male floral buds of the three species of poplar are shown in Table 6.

This variation could be due to genetic differences between the species, influencing their metal uptake mechanisms. White poplar, although slightly lower in metal accumulation overall, shows the highest concentration of lead, which may suggest a different mechanism of lead absorption or a distinct environmental factor affecting this species.

### 2.5. Cytotoxic Effect

The cytotoxic effect of poplar extracts was tested on the fibroblast-like human osteosarcoma MG63 cell line. The MG63 cell line is widely utilized in cytotoxicity studies due to its origin from human osteosarcoma, making it a representative model for osteoblastic cells involved in bone formation and remodeling. These cells exhibit a high proliferation rate, strong adherence to various surfaces, and the ability to synthesize bone matrix-specific proteins, which are essential characteristics for studying osteosarcoma biology. Additionally, MG63 cells demonstrate sensitivity to a broad range of bioactive compounds, including chemotherapeutic agents, making them an effective model for assessing drug-induced cytotoxicity. Their ease of culture and maintenance further enhances their suitability for experimental research, providing a robust and reproducible platform for evaluating the therapeutic potential of novel compounds targeting osteosarcoma.

**Figure 3:**
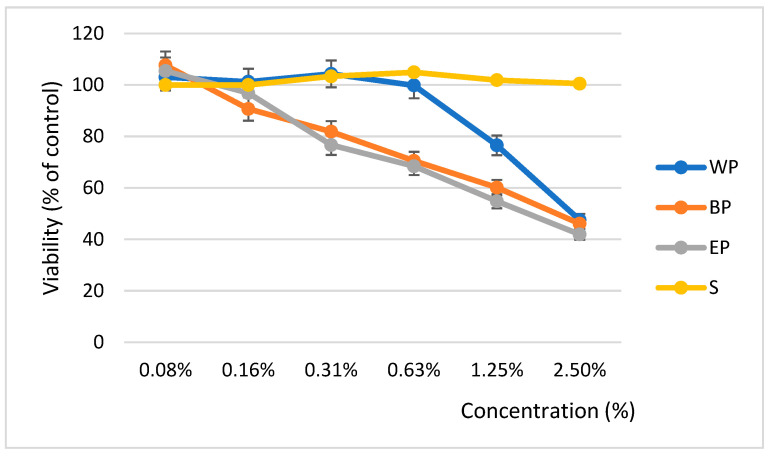
Cytotoxic effects of the three species of *Populus* extracts. S—solvent, EP—Euroamerican poplar, BP—black poplar, WP—white poplar.

CellTiter analysis showed that the percentage of live cells decreased in a concentration-dependent manner. Figure 3 illustrates the effects of poplar extracts on cell viability, which was determined as a percentage compared to the control (untreated cells). The calculated IC_50_ was 132.49 µg/mL for *P. alba*, 42.55 µg/mL for *P. nigra*, 40.87 µg/mL for *P. × euramericana*.

**Figure 4:**
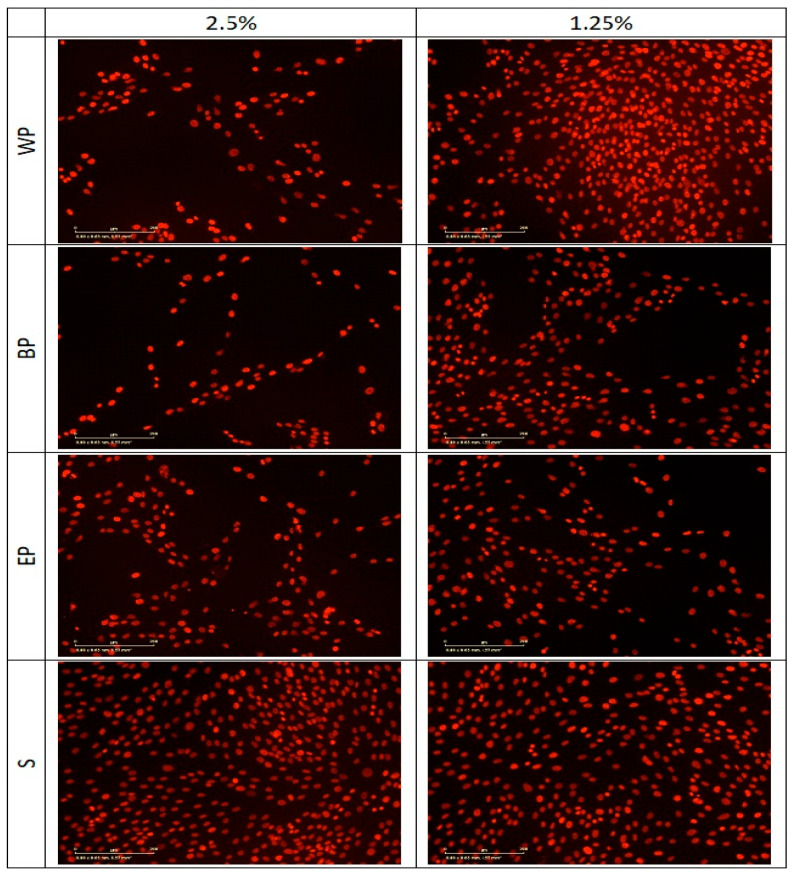
Fluorescence microscopy image revealing the effects of different *Populus* extracts. S—solvent, EP—Euroamerican poplar, BP—black poplar, WP—white poplar. Scalar bar: 2 mm.

Similar results were observed using fluorescence microscopy (Figure 4). Ethanol fixation permeabilizes the cell membrane, allowing propidium iodide (PI) to enter all cells. This method stabilizes the nuclear structure, causing all cell nuclei to appear stained with PI. The intense red fluorescence reflects the concentration of DNA. In addition, nuclear changes, such as chromatin condensation or the formation of apoptotic bodies, can be observed.

Fluorescence microscopy images demonstrate a concentration-dependent effect of extracts obtained from different *Populus* species on cell viability. At a higher extract concentration (2.5%), a visible reduction in cell density and fluorescence intensity is observed, indicating potential cytotoxic effects or inhibited cell proliferation. In contrast, at a lower concentration (1.25%), cells appear more abundant and evenly distributed, suggesting improved survival and metabolic activity. The impact varies depending on the species, with some exhibiting stronger effects than others.

## 3. Discussion

The results demonstrate significant differences in the flavonoid content of the three *Populus* species when extracted with 50% hydroethanolic compared to water, emphasizing the influence of solvent polarity on flavonoid solubility. The hydroethanolic extract (50%) from *P. nigra* showed the highest flavonoid content, suggesting that this solvent is particularly effective in extracting flavones from this species, which may contain a higher proportion of less polar flavonoid compounds. *P. × euramericana* had a slightly lower flavonoid content in hydroethanol, whereas *P. alba* showed the lowest content, indicating interspecific differences in flavonoid composition and extractability. In contrast, flavonoid content in aqueous extracts was significantly lower in all species, probably due to the limited ability of water to dissolve less polar compounds. Interestingly, *P. × euramericana* showed the highest flavonoid content in aqueous extracts, suggesting that it contains a higher proportion of water-soluble flavonoids compared to the other species. In contrast, *P. nigra* and *P. alba* showed a marked reduction in flavonoid content in water, consistent with the higher extractability of flavonoids in hydroethanol. These findings emphasize the impact of solvent choice on extraction efficiency in close correlation with flavonoid solubility depending on the species, which could contribute to the selection of solvents for targeted extraction in applications.

The results also indicate significant differences in total polyphenol content among the three *Populus* species. For 50% hydroethanolic extracts, *P. × euramericana* consistently showed the highest polyphenol content in gallic acid equivalents (33.37 mg/g) and caffeic acid equivalents (33.40 mg/g). *P. nigra* also demonstrated consistently high levels of polyphenols in the 50% hydroethanolic extracts, with slightly lower values than *P. euramericana*. At the same time, *P. alba* exhibited the lowest polyphenol content in both gallic acid and caffeic acid equivalents. Extracts obtained using water as a solvent showed lower polyphenol content compared to 50% hydroethanolic extracts, regardless of the species. Despite this, *P. × euramericana* maintained the highest polyphenol content in both gallic acid equivalents (27.90 mg/g) and caffeic acid equivalents (30.08 mg/g), followed by *P. nigra*, with *P. alba* again showing the lowest content. Interestingly, the order of polyphenol content remained consistent for both extraction methods and reference standards (gallic acid equivalents or caffeic acid equivalents), with *P.× euramericana* exhibiting the highest polyphenol content, followed by *P. nigra*, with *P. alba* consistently having the lowest levels.

The antioxidant activity of the three *Populus* species, evaluated using DPPH and FRAP assays, showed variations depending on species and solvent. Among the species, *P. nigra* demonstrated the highest antioxidant capacity, reflected in significantly lower IC_50_ values and higher FRAP values, indicating a greater concentration of phenolic compounds. *P. × euramericana* exhibited similar but slightly lower activity, while *P. alba* consistently showed the lowest antioxidant activity in both tests. The results revealed that hydroethanolic extracts exhibited superior free radical-scavenging activity compared to that of aqueous extracts, closely correlated with polyphenolic content. These findings align with similar studies on *Populus* species and other plants, where ethanol extraction yielded higher antioxidant activity [47,48]. This observation highlights the influence of solvent choice in enhancing the isolation of phenolic compounds, supporting the hypothesis that phenolics are the primary contributors to antioxidant capacity. Future research focusing on the isolation and characterization of phenolic compounds from these species will provide deeper insights into their antioxidant mechanisms and applications.

The antioxidant activity of polyphenols plays an important role in protecting human health by neutralizing free radicals, which can damage cells and contribute to chronic diseases. By reducing oxidative stress, polyphenols support cardiovascular health, provide neuroprotection, and help lower the production of pro-inflammatory markers. Additionally, they contribute to preventing premature skin aging, highlighting their significant role in maintaining overall health and preventing age-related conditions [8,49].

For all three poplar species, the hydroethanolic extracts exhibited the highest polyphenol contents and demonstrated significant antiradical activities.

The identification of different polyphenolic compounds from the male flower buds of *Populus* species using the HPTLC method highlighted the phytochemical diversity within this genus.

In *P. nigra*, notable phenolic compounds include chlorogenic acid, apigenin, quercetin, caffeic acid, and kaempferol. Chlorogenic acid, an important antioxidant, is recognized for its health benefits, including anti-inflammatory and neuroprotective effects [50,51,52]. Quercetin and kaempferol are associated with free radical-scavenging capacity, which may contribute to the species’ resistance to oxidative stress. *P. × euramericana* displayed a distinct profile, with compounds such as apigenin, isoquercetin, luteolin, and isochlorogenic acid identified. Isoquercetin, a glycosylated form of quercetin, is associated with increased bioavailability and potential health benefits [35,53]. The flavonoid diversity of this hybrid species may reflect its adaptability and successful integration of traits from its parental species, contributing to its widespread use in forestry and bioenergy production. In contrast, *P. alba* exhibited a more limited range of identified compounds, including rutin, chlorogenic acid, apigenin, and quercetin. The presence of rutin, known for its anti-inflammatory and cardiovascular protective properties, suggests specific health benefits.

The concentration of caffeic acid determined by the HPLC method was nearly 10 times higher in the black poplar. This considerable difference suggests that black poplar extract may exhibit higher antioxidant potential, given that caffeic acid is recognized for its antioxidant properties, which play a significant role in mediating anti-inflammatory and anticarcinogenic effects [54].

The concentration of *p*-coumaric acid in the black poplar extract (0.054 mg/mL) was approximately twice as high as that in the Euroamerican poplar extract (0.025 mg/mL). *p*-Coumaric acid is another phenolic compound known for its antioxidant activity and protective health effects, including anti-inflammatory and antitumor properties.

Other phenolic acids identified in the three poplar species from the South of Romania include isoferulic acid, 3,4-dimethoxycinnamic acid, 5-O-coumaroyl-D-quinic acid, and 4-methoxycinnamic acid. These phenolic acids contribute to the antioxidant activity of the three species of poplar, along with various other biological effects, including anticancer [55,56,57], analgesic [58], immunomodulatory, and anti-inflammatory properties [59]. Notably, the Euroamerican poplar species contains flavonoids (4′-hydroxyflavanone and genistein) and tannins (catechin), which provide a unique phytochemical profile for this species. The diverse pharmacological actions of flavonoids are well-documented in many studies, including antioxidant and protective effects, as well as anticancer, antiallergic, diuretic, anxiolytic, and antimicrobial activities [60,61,62,63,64].

5-Hydroxypipecolic acid (5-HPA), detected in white poplar and identified as a 3TMS (trimethylsilyl) derivative using gas chromatography-mass spectrometry (GC-MS), is a compound of interest due to its significant biological and chemical properties. 5-Hydroxypipecolic acid is often associated with plant defense mechanisms. This highlights the potential importance of this compound in the adaptive strategies of this tree species, especially under harsh environmental conditions or against pests and pathogens. Furthermore, 5-hydroxypipecolic acid, a hydroxylated derivative of pipecolic acid, plays an important role in pharmaceuticals and chemicals as an intermediate in the synthesis of drugs, including β-lactamase inhibitor antibiotics such as avibactam, and as a marker for neurological degenerative disorders [65].

Numerous studies have investigated the chemical composition of various *Populus* species, revealing a rich diversity of polyphenolic compounds. The present findings are consistent with those reported by Rubiolo et al. [14,66], who identified hydroxycinnamic acids, salicylate glycosides, and flavonoids such as pinocembrin and chrysin using methods like GC-MS and HPLC-PDA. Additionally, aldehyde derivatives like salicylaldehyde, derived from salicin or populin, were noted, consistent with the results of Jerković and Mastelić [16]. Ristivojević et al. identified a diverse array of phenolic compounds in Serbian ethanolic extracts, including phenolic acids, flavan-3-ols, and glycosides [9]. In contrast, Dudonné et al. found that aqueous extracts from Bulgaria contained specific flavonoids (like pinocembrin) and salicin [10]. Further, studies in Algeria demonstrated that methanol and ethyl acetate extractions yielded different phenolic compositions, with unique compounds like rosmarinic acid appearing in methanol extracts. Greenaway et al. also noted geographic variability in phenolic content among samples from different countries, with UK specimens particularly rich in flavones like chrysin and galangin [67].

The large amounts of sugars found in the three species of poplar are related to their important physiological role in providing energy, serving as a foundation for growth and development during various biological phases. Moreover, these sugars, particularly sucrose, act as a trigger for flowering, serving as a primary signal for the maturation of flower buds [68]. The high concentration of sucrose in the male buds of white poplar (32.79%) indicates that these buds were close to opening and releasing pollen grains through the stamens. In contrast, the male buds of black poplar and Euroamerican poplar were in an earlier stage of opening, as indicated by their lower sucrose levels (15.67% and 10.01%, respectively).

To ensure the safety of plant-based materials for human health, we determined of heavy metal content, focusing on potential environmental contamination. By confirming that the levels of harmful metals fall within acceptable limits, we can assure that the plant materials pose no significant risk to human health, in accordance with global safety guidelines and consumer protection standards [69,70].

The determination of heavy metals in the analyzed poplar samples confirms their safety for use in pharmaceutical and cosmetic applications, as the concentrations of Hg, Cd, Pb, Cu, and Ni were within the permissible limits established by regulatory standards for plant materials. The low levels of Cd and Pb suggest minimal environmental contamination, while the measured levels of Cu and Ni, which are essential trace elements, remained well below toxic thresholds. These results underscore the safety and suitability of the poplar extracts and highlight the importance of periodic toxic metal analyses to ensure compliance with safety guidelines and minimize health risks [71,72,73,74,75,76,77].

In this study, extracts of *P. nigra* and *P. × euramericana* exhibited significant cytotoxic activity against fibroblast-like human osteosarcoma MG63 cells, with IC_50_ values of 42.55 µg/mL for *P. nigra*, 40.87 µg/mL for *P. × euramericana*, and 132.49 µg/mL for *P. alba*. According to the American National Cancer Institute, extracts from these three *Populus* species from southern Romania demonstrate moderate cytotoxicity, with IC_50_ values below 200 µg/mL [78]. These IC_50_ values are consistent with previous findings, such as those reported by Kis et al. (2022), who observed a similar efficacy of *P. nigra* bud extracts in MCF-7 breast cancer cells, achieving an IC_50_ of 66.26 µg/mL [8].

Future studies should focus on isolating and characterizing the specific bioactive compounds in these extracts that are most responsible for the observed cytotoxic effects, paving the way for the development of therapeutic agents derived from natural compounds. *P. alba* exhibited the highest IC_50_ value, indicating the weakest cytotoxic effect among the three species. In contrast, the lower IC_50_ values for *P. nigra* and *P. × euramericana* suggest a higher concentration or potency of bioactive compounds, such as polyphenols, which are well-documented for their cytotoxic and antioxidant properties. Further phytochemical investigations are essential to identify and quantify the specific compounds contributing to the observed effects and to elucidate the mechanisms of action. In addition, assessment of the selectivity of these extracts in terms of their effects on the viability of normal versus cancer cells could provide essential information on their therapeutic potential.

## 4. Materials and Methods

### 4.1. Plant Material

**Figure 5:**
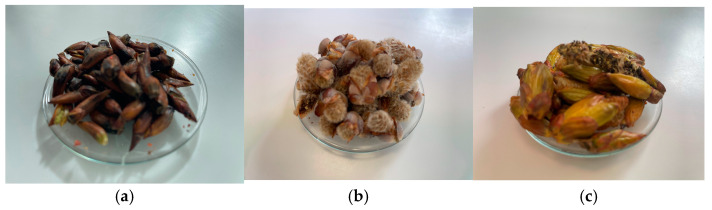
Male floral buds of *Populus* species: (**a**) *P. nigra* L.; (**b**) *P. alba* L.; and (**c**) *P.* × *euramericana* (Dode) Guinier.

For this study, fresh male floral buds were collected from forests at the edge of Oinacu Village, Giurgiu County, in the South of Romania, in March 2024 (Figure 5). Voucher specimens of the three species—*Populus nigra* L., *Populus alba* L. and *Populus* × *euramericana* (Dode) Guinier—were deposited at the University of Bucharest Herbarium in the “Dimitrie Brândză” Botanical Garden with numbers 410485, 410486, and 410487.

### 4.2. Plant Extracts

Poplar buds from the three species were air dried in the shade at room temperature (25 °C) for 20 days, and ground in a mechanical grinder. Extractions were performed by refluxing 1 g of plant material with 100 mL of solvent for 30 min using a water bath. Purified water was used for the aqueous extracts, while 50% (*w*/*w*) ethanol was used for the hydroethanolic extracts. The extracts were filtered using ashless filter paper (ash content: 0.007%, diameter: 90 mm, retention: 8–12 µm). Subsequently, all samples were adjusted to a standardized final volume and stored at 5 °C in a refrigerator.

### 4.3. Spectrophotometric Determination of Total Flavonoids and Total Polyphenols

#### 4.3.1. Total Flavonoid Content (TFC)

For the determination of total flavonoid content, the spectrophotometric method with aluminum chloride in the presence of sodium acetate was used, as described in the 10th edition of the Romanian Pharmacopoeia, where the absorbance of the yellow complex formed allows for the quantification of total flavonoids [79]. Ten milliliters (10 mL) of hydroethanolic and aqueous extracts of the three poplar bud species were diluted with methanol in 25 mL volumetric flasks and filtered. To 5 mL of the diluted extract solutions, 5 mL of 100 g/L sodium acetate solution (Sigma Chemical, St. Louis, MI, USA) and 3 mL of 25 g/L aluminum chloride solution (Merck Group, Darmstadt, Germany) were added, and then the solution was topped with methanol in 25 mL volumetric flasks and the mixture was homogenized. The samples were incubated for 15 min at room temperature, and absorbance was measured at 430 nm using a VWR UV-6300 PC spectrophotometer (VWR International, Wien, Austria). A calibration curve prepared with rutin (Sigma Chemical, St. Louis, MI, USA) was used for quantification. The results were expressed as mg rutin equivalent per gram dry weight (mg RE/g DW). Measurements were carried out in triplicate.

#### 4.3.2. Total Polyphenolic Content (TPC)

The total polyphenolic content of poplar bud extracts was determined spectrophotometrically using Folin–Ciocalteu reagent with gallic acid and caffeic acid as reference standards for quantification [46,80].

To 1 mL poplar bud hydroethanolic or aqueous extract, 4.5 mL deionized water and 2.5 mL of diluted Folin–Ciocalteu reagent (Sigma Chemical, St. Louis, MI, USA) were added. To this mixture, 2 mL of 7% sodium carbonate was added after 5 min. The reaction samples were incubated for 30 min at room temperature, and the absorbance was measured at 765 nm with a VWR UV-6300 PC spectrophotometer (VWR International, Wien, Austria). The total polyphenolic contents of the extracts was determined using calibration curves for gallic acid and caffeic acid (Sigma Chemical, St. Louis, MI, USA). The results were expressed as mg gallic acid equivalent per g of dry weight (mg GAE/g DW) and in mg caffeic acid equivalent per g dry weight (mg CAE/g DW). The experiments were performed in triplicate.

### 4.4. Antioxidant Capacity

#### 4.4.1. DPPH-Scavenging Capacity

The antioxidant activity of the extracts was evaluated using the DPPH (2,2-diphenyl-1-picrylhydrazyl) radical-scavenging method, one of the most popular methods for evaluating antioxidant capacity [81,82]. The method is based on the reduction of the DPPH radical, which leads to a color change from violet to yellow. This color change was monitored by measuring the absorbance at 515 nm after a reaction time of 30 min compared to that of a control containing ethanol using a VWR UV-6300 PC spectrophotometer (VWR International, Wien, Austria). The percent inhibition of DPPH radical activity was calculated using the following formula [83]:

(1) $$DPPH\;inhibition\;\left(\%\right)=(Absorbance\;of\;control-Absorbance\;of\;control)/Absorbance\;of\;tested\;sample)\times 100$$

The antioxidant activity of the samples was expressed as an IC_50_ value, which represents the concentration of sample required to reduce DPPH radical activity by 50%.

#### 4.4.2. FRAP Antioxidant Capacity

The antioxidant capacity of the extracts obtained from the three Romanian poplar species was also evaluated using a FRAP assay, which measures the ability of the antioxidants present in the sample to reduce ferric ions (Fe^3+^) to ferrous ions (Fe^2+^). The reduction process leads to the formation of a blue-colored ferrous–tripyridyltriazine (Fe^2+^-TPTZ) complex, which can be quantified by the spectrophotometric measurement of its absorbance at 593 nm using a VWR UV-6300 PC UV-Vis spectrophotometer (VWR International, Wien, Austria) [81]. A calibration curve was prepared using ferrous sulfate standards (FeSO_4_·7H_2_O) at concentrations ranging from 100 to 1000 μmol/L. All measurements were carried out in triplicate.

### 4.5. TLC Identification of Polyphenols

The identification of phenolic acids and flavonoid compounds by thin-layer chromatography was performed using an HPTLC system (CAMAG, Muttenz, Switzerland) consisting of a Linomat 5 applicator, an ADC 2 development chamber, a TLC visualizer 2, and winCATS, version 2.4. and VideoScan Evaluation Software, version 028.2200. For the identification of phenolic acids and flavonoids in plant extracts using HPTLC, the following reference substances were employed: rutin, isoquercetin (quercetin-3-glucoside), rosmarinic acid, caffeic acid, chlorogenic acid, isochlorogenic acid, *p*-coumaric acid, ferulic acid, apigenin-7-glucoside, apigenin, kaempferol-3-glucoside, kaempferol, luteolin-7-glucoside, and luteolin.

The stock reference solutions were prepared by dissolving the compounds of interest in methanol at concentrations of 1 mg/mL. Several mixed reference solutions were prepared by mixing and dilution in methanol.

For thin-layer chromatography (TLC) analysis, extracts were diluted 1:10 (*v*/*v*) in methanol, ultrasonicated for 5 min, and centrifuged at 5000 rpm for 10 min at 4 °C. From the resulting solutions, aliquots of 2 μL of the reference solutions and 2 μL and 4 μL of the test solutions, respectively, were applied to Silicagel 60 F254 plates, size 20 × 10 cm. The migration was performed with two methods: (1) using a mobile phase composed of ethyl acetate/formic acid/acetic acid/water at a ratio of 100:11:11:26 (*v*/*v*/*v*/*v*) over a distance of 7 cm, and (2) using a mobile phase composed of hexane/ethyl acetate/formic acid at a ratio of 18:20:2 (*v*/*v*/*v*) over a distance of 8 cm. After migration, the plates were oven dried at 100–105 °C. Derivatization was performed by spraying a 10 g/L solution of 2-aminoethyl diphenylborate in methanol R, followed by the application of a 50 g/L solution of polyethylene glycol 400 R in methanol. After drying in air for 30 min, the plates were read under white light at a wavelength of λ = 366 nm, and chromatogram processing was performed with the equipment software [84].

### 4.6. HPLC-DAD Identification and Quantification of Polyphenolic Compounds

*Populus* spp. extracts were analyzed by HPLC using an Agilent Technologies 1260 series system (Santa Clara, CA, USA) equipped with a DAD detector, binary pump, thermostatted autosampler, degasser, and column thermostat. Chromatographic separation was performed on a Zorbax Eclipse C18 column (Agilent, Santa Clara, CA, USA), 150 mm × 3 mm, 3.5 µm, at 35 °C, with a mobile phase of methanol/acetonitrile/water (45:45:10 *v*/*v*/*v*) and 0.1% phosphoric acid at an initial ratio of 23:77 (*v*/*v*), a flow rate of 0.5 mL/min, and a linear gradient elution from 35% to 65% methanol over 25 min. The chromatographic signals at different wavelengths of 254 nm and 340 nm were simultaneously monitored for the detection of the compounds.

Caffeic acid, p-coumaric acid, isochlorogenic acid, quercetin-3-glucoside (isoquercetin), quercetin, apigenin, kaempferol-3-glucoside, kaempferol, luteolin-7-glucoside, and luteolin were used as reference substances.

The reference substances (caffeic acid, p-coumaric acid, isochlorogenic acid, quercetin-3-glucoside, quercetin, apigenin, kaempferol-3-glucoside, kaempferol, luteolin-7-glucoside, and luteolin) were dissolved in methanol to prepare stock solutions, each with a concentration of 1 mg/mL. A mixed standard solution was prepared from the stock solution. In this solution, each compound was present at a concentration of 10 µg/mL, except for p-coumaric acid, which had a 20 µg/mL concentration. The poplar extracts were diluted at a ratio of 1:10 (*v*/*v*) in 50% methanol. The diluted samples were subjected to ultrasonication for 5 min. After ultrasonication, the samples were centrifuged at 5000 rpm for 10 min at 4 °C. Once centrifuged, a portion of the clarified solution was filtered through a 0.45 µm PTFE filter directly into an autosampler vial. Compounds in the extract samples were identified by comparing their retention times with those of the standard substances. Beyond retention times, the UV spectra of the compounds were also compared with those of the standards for additional verification. Mass Hunter software, version 3.1.199 was used [15,85]. Chromatograms were recorded at a wavelength of 305 nm.

A standard solution containing 100 µg/mL caffeic acid and 50 µg/mL p-coumaric acid was prepared to quantify of the reliably identified compounds. From this solution, calibration solutions were prepared at the following concentration levels: 5, 10, 20, and 50 µg/mL for caffeic acid and 2, 5, 10, and 25 µg/mL for p-coumaric acid.

### 4.7. GC-MS/MS Identification of Compounds from Poplar Species Extracts

A chromatographic screening of the compounds from the male floral buds of Populus species was performed using a Thermo Scientific system (Waltham, MA, USA) consisting of GC TRACE 1310, a TriPlus TSH autosampler, and a TSQ EVO8000 triple quadrupole mass spectrometer assisted by Chromeleon software Version 7.2.10 and the NIST mass spectra library. For the analysis, 50 µL of the extract was evaporated to dryness under a nitrogen stream, and the resulting residue was derivatized using BSTFA. The GC-MS-MS screening was carried out using a 30 m HP-5MS column under the following analysis conditions: flow rate—1 mL/min, injection volume—1 µL, injector temperature—280 °C, and splitting rate—60, with the temperature program of the oven starting from 100 °C with a gradient of 10 °C/min up to 280 °C [25,86]. The identification of compounds by the GC-MS-MS technique was performed using the NIST version 2.2 library.

### 4.8. Heavy Metal Detection

#### 4.8.1. Mercury Analysis

The mercury (Hg) concentration was determined using a Milestone DMA-80 direct mercury analyzer (Milestone Srl, Sorisole, Bergamo, Italy). The poplar samples were incinerated at 650 °C to release Hg vapors, which were captured in a gold amalgamator and then desorbed for detection (at 260 nm) through atomic absorption spectroscopy [87]. The accuracy of the mineral composition analysis of the samples was validated using the certified reference material IAEA-140-OC [88] from the International Atomic Energy Agency (Vienna, Austria). All analyses were conducted in triplicate. The analytical method used to measure mercury (Hg) concentration in the certified reference material (IAEA-140-OC) demonstrates high precision and acceptable accuracy. The mean measured value for the Hg concentration was 0.0342 ± 0.0002 mg/kg, with a small standard deviation indicating minimal variability in the measurements. The relative standard deviation (RSD) of 0.58% further confirms the method’s excellent repeatability across triplicate measurements. When compared to the certified value of 0.038 mg/kg, the measured value shows a slight deviation but remains within an acceptable range. Additionally, the percent recovery of 90% suggests the method effectively captures the true concentration of Hg, affirming its reliability for analytical purposes.

#### 4.8.2. Heavy Metal Analysis

Metal concentrations were determined using an inductively coupled plasma mass spectrometer, ICP-MS Plasma Quant Elite (Analytik Jena, Jena, Germany). A speedwave XPERT/XTRACT (Berghof, Germany) microwave digestion dish was used to digest the samples. The reagents HNO_3_ and HCl were obtained from Merck (Darmstadt, Germany), and the reference material IAEA-140-OC from the International Atomic Energy Agency. For the preparation of measurement solutions, 0.5 g of each sample was weighed and individually transferred into a speedwave XPERT/XTRACT microwave digestion vessel (Berghof, Eningen, Germany). The dried material from each poplar sample (0.5 g) was diluted with 8 mL of concentrated nitric acid (HNO_3_) and 1 mL of concentrated hydrochloric acid (HCl) at a temperature of 300 °C for approximately 1 h. The digested samples were then mixed with distilled water and filtered using 0.45 μm Whatman filter paper. After cooling, the filtered solutions were transferred into 50 mL volumetric flasks and topped up to the mark with distilled water [89]. Two reference materials were used: ERM-CD 200 [90] from the European Commission, Institute for Reference Materials and Measurements (IRMM) (Geel, Belgium), and BCR-414 [91] from the European Commission, Joint Research Centre Ispra, Italy. The reference materials were used, in accordance with the previously mentioned analytical methodology, to verify complete digestion and confirm the recovery of the analyte. ERM-CD200 is extracted from the brown algae Bladderwrack (*Fucus vesiculosus*) and is a certified reference material containing trace elements such as As, Cd, Cu, Hg, Pb, Se and Zn. BCR-414 is made from freeze-dried plankton powder and provides certified key trace elements, including As, Cd, Cr, Cu, Fe, Hg, Ni, Mn, Pb, Se, V, and Zn [92]. The digested solutions were analyzed using inductively coupled plasma mass spectrometry (ICP-MS). Table 7 presents the certified concentrations, measured concentrations, and recovery rates of the two reference samples: ERM-CD200 and BCR-414. The values obtained for the recovery percentage (89.5–106.7%) demonstrate the high precision of the analytical method used.

### 4.9. Cytotoxic Effect

The CellTiter 96^®^ AQueous One Solution Cell Proliferation Assay (Promega, Madison, WI, USA) was used to evaluate the cytotoxicity of the extracts of the three Romanian species of *Populus*. The fibroblast-like human osteosarcoma MG63 cell line was purchased from the American Type Culture Collection (ATCC^®^ CRL-1427™, Manassas, VA, USA) and seeded in 96-well culture plates in DMEM:F12 medium (Sigma, USA) supplemented with 10% fetal bovine serum (Sigma, USA) at a concentration of 5 × 10^3^ cells/well. After recovery, cells (passage number 111) were treated with *Populus* extracts prepared in a series of binary dilutions. A stock solution of 1000 µg/mL (*w*/*v*) was initially prepared by diluting the obtained extracts. From this stock solution, serial binary dilutions were performed for biological assays, yielding the following final concentrations: 2.5% (*v*/*v*), 1.25% (*v*/*v*), 0.63% (*v*/*v*), 0.32% (*v*/*v*), 0.16% (*v*/*v*), and 0.08% (*v*/*v*).

At 24 h, a reagent containing a tetrazolium compound [3-(4,5-dimethylthiazol-2-yl)-5-(3-carboxymethoxyphenyl)-2-(4-sulfophenyl)-2H-tetrazolium, inner salt; MTS] and an electron coupling reagent (phenazine ethosulfate; PES) was added to each sample in the plates. After adding the CellTiter reagent, the plates were shaken to ensure the even distribution of the reagent and incubated at 37 °C for 4 h. During this time, the cells bio-reduced the added tetrazolium salt into a soluble formazan. The product was measured spectrophotometrically at 490 nm using a TriStar LB 942 multimodal microplate reader (Berthold Technologies, Bad Wildbad, Germany). The IC_50_ Calculator (“Quest Graph™ IC_50_ Calculator”. AAT Bioquest, Inc. (Pleasanton, CA, USA), https://www.aatbio.com/tools/ic50-calculator (accessed on 4 July 2024)) was used to establish the concentration at which each *Populus* extract exerted half of its maximal inhibitory effect.

The second method for evaluating *Populus* cytotoxicity was microscopy. The reduction in cells was highlighted by fixing the cells with 70% ethanol and staining them with 5 μg/mL propidium iodide, Images were captured using an Axio Observer D inverted microscope with a fluorescence module (Carl Zeiss, Munich, Germany).

### 4.10. Statistical Data Processing

Statistical analysis of the data was carried out using XL STAT 2022.4.5 statistics software by calculating the mean and standard deviation (±SD) and performing an analysis of variance (ANOVA). All the experiments in the study were realized in triplicate.

## 5. Conclusions

This study highlights the significant polyphenolic diversity, antioxidant activity, and cytotoxic potential of the male buds from three *Populus* species (*P. nigra*, *P. × euramericana*, and *P. alba*) growing in southern Romania. The phytochemical analysis revealed a rich composition of polyphenols, with *P. nigra* containing chlorogenic acid, caffeic acid, quercetin, and kaempferol, which are associated with strong antioxidant and health-protective effects. *P. × euramericana* exhibited a unique profile with isoquercetin, luteolin, and catechins, reflecting the hybrid’s vigor, while *P. alba* contained rutin and 5-hydroxypipecolic acid, indicating distinct bioactive properties.

The choice of solvent significantly influenced extraction efficiency, with 50% hydroethanolic extracts yielding higher flavonoid and phenolic acids content compared to aqueous extracts. This higher polyphenolic content contributed to the higher antioxidant activity observed in hydroethanolic extracts compared to aqueous extracts. Among the species tested, *P. nigra* exhibited the strongest antioxidant activity, followed by *P. × euramericana* and *P. alba*, reflecting their respective phenolic contents.

Cytotoxicity assessments revealed the potential of *Populus nigra* and *Populus × euramericana* extracts as therapeutic agents against fibroblast-like human osteosarcoma MG63 cell lines, eliciting a concentration-dependent response to all tested species. Future research incorporating normal cell models will be essential to assess the extracts’ safety and selectivity more comprehensively. The results of this study indicate a direct correlation between polyphenol content, antioxidant activity, and cytotoxic effects in the three *Populus* species. *P. nigra* and *P. × euramericana*, which exhibited the highest polyphenol contents, also demonstrated the strongest antioxidant activities and cytotoxic effects, suggesting that these bioactive compounds may be responsible for both the antioxidant properties and the observed cytotoxic potential.

Further research should focus on isolating and characterizing specific bioactive compounds to elucidate their mechanisms of action and to assess other pharmacological uses for these valuable species.

## Figures and Tables

**Table 1:** Total polyphenolic and flavonoid contents in male buds of three *Populus* species.

Plant Species	Solvents	TFC (mg RE/g DW)	TPC (mg GAE/g DW)	TPC (mg CAE/g DW)
*P. nigra*	50% HE Water	4.45 ± 0.337 2.79 ± 0.116	32.83 ± 1.775 23.83 ± 0.964	32.91 ± 1.799 21.99 ± 0.902
*P. alba*	50% HE Water	2.94 ± 0.187 2.15 ± 0.077	20.92 ± 0.880 19.26 ± 0.795	19.26 ± 0.803 19.62 ± 0.855
*P.* × *euramericana*	50% HE Water	3.93 ± 0.305 3.07 ± 0.285	33.37 ± 1.851 27.90 ± 1.053	27.90 ± 1.120 30.08 ± 1.572

TFC—total flavonoid content (mg rutin/g DW); TPC—total polyphenolic content (mg gallic acid/g DW and mg caffeic acid/g DW).

**Table 2:** Retention factor (R_f_) values of reference compounds used for qualitative HPTLC analysis of populus extracts.

	Mobile Phase 1 (Figure 1a)		Mobile Phase 2 (Figure 1b)	
Compounds	Track	R_f_	Track	R_f_
Rutin	1	0.37	1	0.02
Chlorogenic acid		0.48		0.03
Apigenin-7-glucoside		0.65		0.06
Apigenin		0.97		0.69
Isoquercetin	2	0.57	2	0.04
Kaempfetol-3-glucoside		0.65		0.05
Kaempferol		0.98		0.75
Luteolin-7-glucoside	3	0.59	3	0.03
Luteolin		0.94		0.55
Caffeic acid	4	0.92	9	0.58
Rosmarinic acid		0.88		0.25
Isochlorogenic acid	5	0.84	10	0.08
Quercetin		0.96		0.62
Ferulic acid	6	0.94	11	0.67
*p*-Coumaric acid		ND		ND

ND—not detected.

**Table 3:** Retention times of compounds identified by HPLC.

Peak Name	Retention Time (min)	Relative Area (%)
Sample name: *P. nigra*		
Caffeic acid	4.625	11.60
*p*-Coumaric acid	8.106	4.24
Luteolin-7-glucoside	10.134	43.06
Caffeic acid derivative	11.134	2.12
Ferulic acid derivative	13.615	28.51
*p*-Coumaric acid derivative	16.502	6.46
Sample name: *P. alba*		
Quercetin glycoside	7.985	11.51
Kaempferol glycoside	9.772	61.28
Sample name: *P.* × *euramericana*		
Caffeic acid	4.633	3.11
*p*-Coumaric acid	8.119	6.45
Kaempferol glucoside	9.796	21.81
Ferulic acid derivative	13.628	4.71
Apigenin	17.910	4.63
Kaempferol	18.112	2.49

**Table 4:** Main components identified by GC-MS in male buds of *P. nigra* L., *P. alba* L., and *P. × euramericana* and derivatized with TMS.

Peak Name	Phytochemical Class	Retention Time (min)	Relative Area (%)
**Sample name: *P. nigra***			
**Benzoic acid, TMS**	Organic acids	8.40	1.29
**Phosphoric acid, tris-TMS**	Mineral acids	8.72	2.15
**Glyceric acid, 3TMS**	Organic acids	9.70	0.37
**Malic acid, 3TMS**	Organic acids	12.24	2.12
**Fructofuranose, 5TMS**	Carbohydrates	18.73	3.33
**D-Fructose, 5TMS**	Carbohydrates	18.81	0.85
**Citric acid, 4TMS**	Organic acids	18.92	0.34
**4-Methoxycinnamic acid, TMS**	Phenolic acids	19.34	0.62
**5-*O*-Coumaroyl-D-quinic acid**	Phenolic acids	19.64	0.37
**α-D-Mannopyranose, 5TMS**	Carbohydrates	20.27	1.20
**Gulonic acid 1,4-lactone, 4TMS**	Carbohydrate derivates	20.34	1.24
**4-Coumaric acid, 2TMS**	Phenolic acids	21.18	0.62
**D-Glucose, 5TMS**	Carbohydrates	21.65	2.46
**Gluconic acid, 6TMS**	Carbohydrate derivates	21.95	7.22
**3,4-Dimethoxycinnamic acid, TMS**	Phenolic acids	22.49	4.03
**Isoferulic acid, 2TMS**	Phenolic acids	23.16	10.26
**Myo-inositol, 6TMS**	Polyols	23.19	1.80
**Caffeic acid, 3TMS**	Phenolic acids	23.88	3.21
**Sucrose, 8TMS**	Carbohydrates	29.01	15.67
**Sample name: *P. alba***			
**Phosphoric acid, tris-TMS**	Mineral acids	8.72	2.09
**Glyceric acid, 3TMS**	Organic acids	9.70	0.34
**Fumaric acid, 2TMS**	Organic acids	10.09	0.27
**Malic acid, 3TMS**	Organic acids	12.25	2.74
**L-Threonic acid, 3TMS**	Carbohydrate derivates	13.14	0.26
**L-(+)-Tartaric acid, 4TMS**	Organic acids	14.19	0.79
**5-Hydroxypipecolic acid, 3TMS**	Organic acids	14.25	0.59
**Fructofuranose, 5TMS**	Carbohydrates	18.73	3.29
**D-Fructose, 5TMS**	Carbohydrates	18.81	0.49
**β-D-(+)-Mannopyranose, 5TMS**	Carbohydrates	20.27	1.36
**Dulcitol, 6THS**	Carbohydrate derivates	20.86	5.75
**D-Glucose, 5TMS**	Carbohydrates	21.65	3.06
**D-Gluconic acid, 6TMS**	Carbohydrate derivates	21.95	1.02
**Myo-inositol, 6TMS**	Polyols	23.20	1.40
**Sucrose, 8TMS**	Carbohydrates	29.01	32.79
**Sample name: *P. × euramericana***			
**Benzoic acid, TMS**	Organic acids	8.40	0.65
**Phosphoric acid, tris-TMS**	Mineral acids	8.72	5.95
**Glyceric acid, 3TMS**	Organic acids	9.70	0.44
**Malic acid, 3TMS**	Organic acids	12.24	2.92
**Tartaric acid, 4TMS**	Organic acids	14.19	0.78
**Xylitol, 5TMS**	Polyols	16.64	1.15
**Fructofuranose, 5TMS**	Carbohydrates	18.72	5.24
**D-Fructose, 5TMS**	Carbohydrates	18.81	1.62
**Citric acid, 4TMS**	Organic acids	18.91	0.55
**5-O-Coumaroyl-D-quinic acid, 5TMS**	Phenolic acids	19.64	0.37
**α-D-Mannopyranose, 5TMS**	Carbohydrates	20.26	1.17
**4-Coumaric acid, 2TMS**	Phenolic acids	21.18	0.34
**D-Glucose, 5TMS**	Carbohydrates	21.65	2.89
**D-Gluconic acid, 6TMS**	Carbohydrate derivates	21.94	3.03
**Myo-inositol, 6TMS**	Polyols	23.20	1.37
**Caffeic acid, 3TMS**	Phenolic acids	23.88	0.56
**Sucrose, 8TMS**	Carbohydrates	29.01	10.01
**4′-Hydroxyflavanone, TMS**	Flavonoids	29.39	2.60
**Genistein, 3TMS**	Flavonoids	30.60	3.88

**Table 5:** Mean ± SD values of Hg concentration in poplar samples.

No.	Sample	Hg Concentration Mean ± SD (mg/kg)
1	*P. alba*	0.0082 ± 0.0001
2	*P*. *nigra*	0.0055 ± 0.0001
3	*P.* × *euramericana*	0.0066 ± 0.0002

SD—standard deviation.

**Table 6:** Correlation coefficients of the calibration curves and average values of metal concentrations, measured in triplicate.

Metal	White Poplar Measured Values (mg/kg)	Black Poplar Measured Values (mg/kg)	Euroamerican Poplar Measured Values (mg/kg)	R^2^
Cd	0.0683	0.3394	0.1038	0.9998
Cu	7.5371	9.7663	8.9777	0.9999
Ni	5.0785	5.6548	6.8684	0.9998
Pb	0.1750	0.1002	0.0624	0.9997

**Table 7:** Comparison between measured and certified values of element concentrations in standard reference materials.

Reference Material	Element	Measured Values (mg/kg)	Certified Values (mg/kg)	Percent Recovery Values (%)
ERM-CD200	Cd	0.85	0.95	89.5
BCR-414	Cu	32	30	106.7
	Ni	18.3	18.8	97.4
	Pb	3.6	3.9	92.3

## Data Availability

Data are contained within the article and Supplementary Materials.

## Supplementary Materials

The following supporting information can be downloaded at: https://www.mdpi.com/article/10.3390/molecules30040913/s1, Figure S1: GC-MS chromatogram of *P. nigra*; Figure S2: GC-MS chromatogram of *P. alba*; Figure S3: GC-MS chromatogram of *P* × *euramericana*.
