# Supplementary material for: Polyphenolic Composition, Antioxidant Activity, and Cytotoxic Effect of Male Floral Buds from Three Populus Species Growing in the South of Romania
Source: Molecules. 2025 Feb 16;30(4):913. doi: 10.3390/molecules30040913 (PMC11857894; doi:10.3390/molecules30040913)
Supplement: Supplementary file 1 [file molecules-30-00913-s001.zip › molecules-3429281-supplementary.pdf]

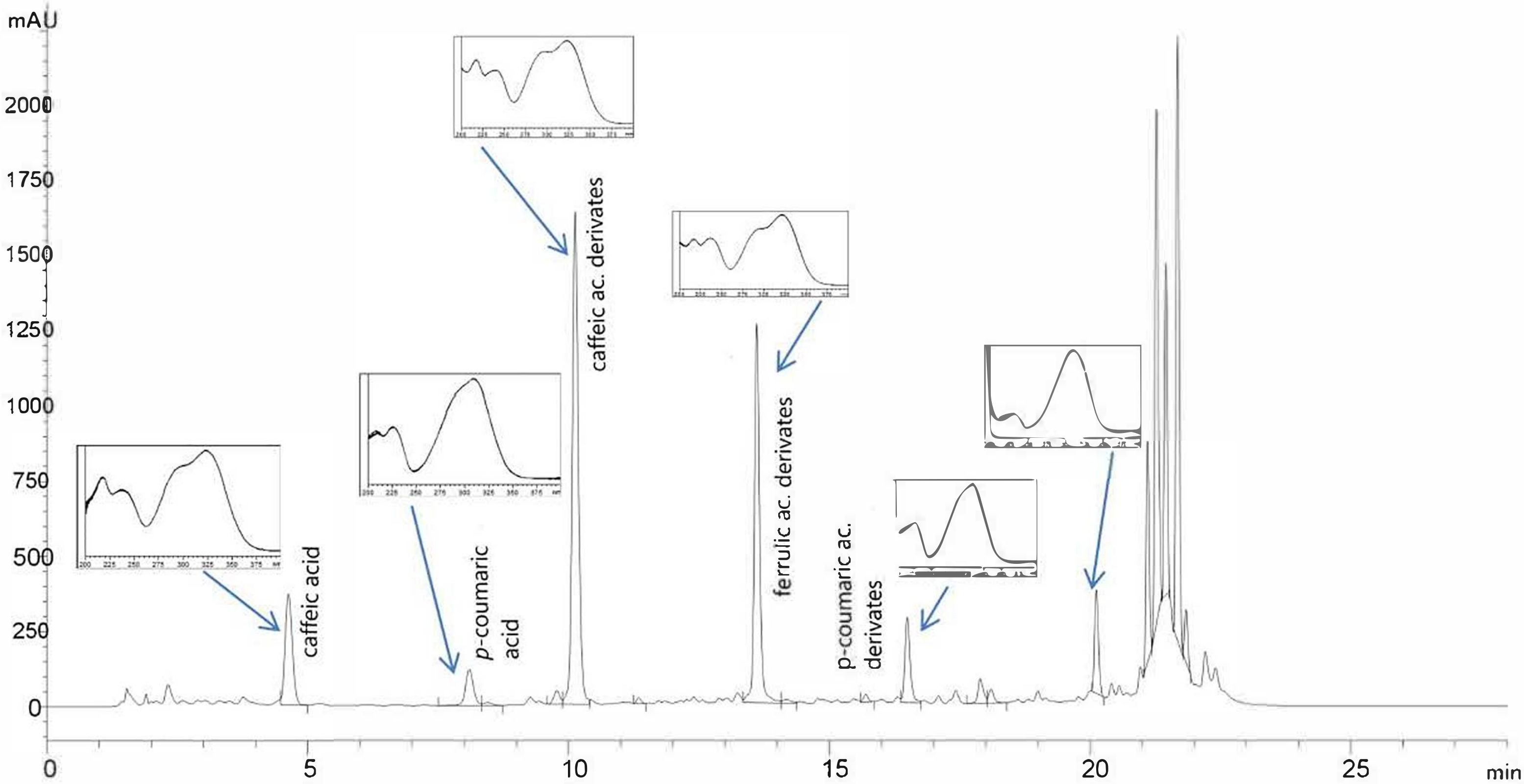

Figure S1: GC-MS chromatogram of *P. nigra*.

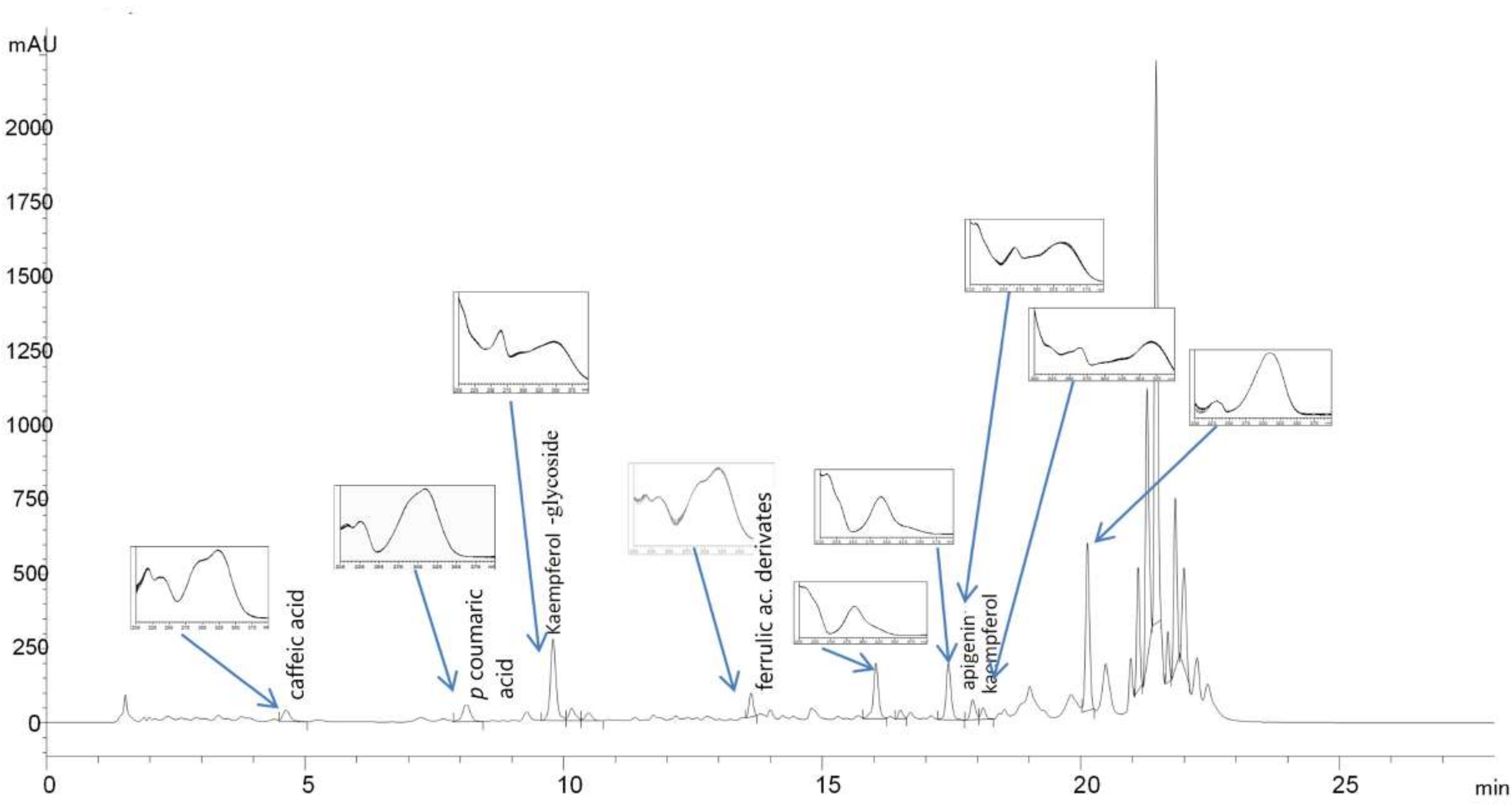

Figure S2: GC-MS chromatogram of *P. alba.*

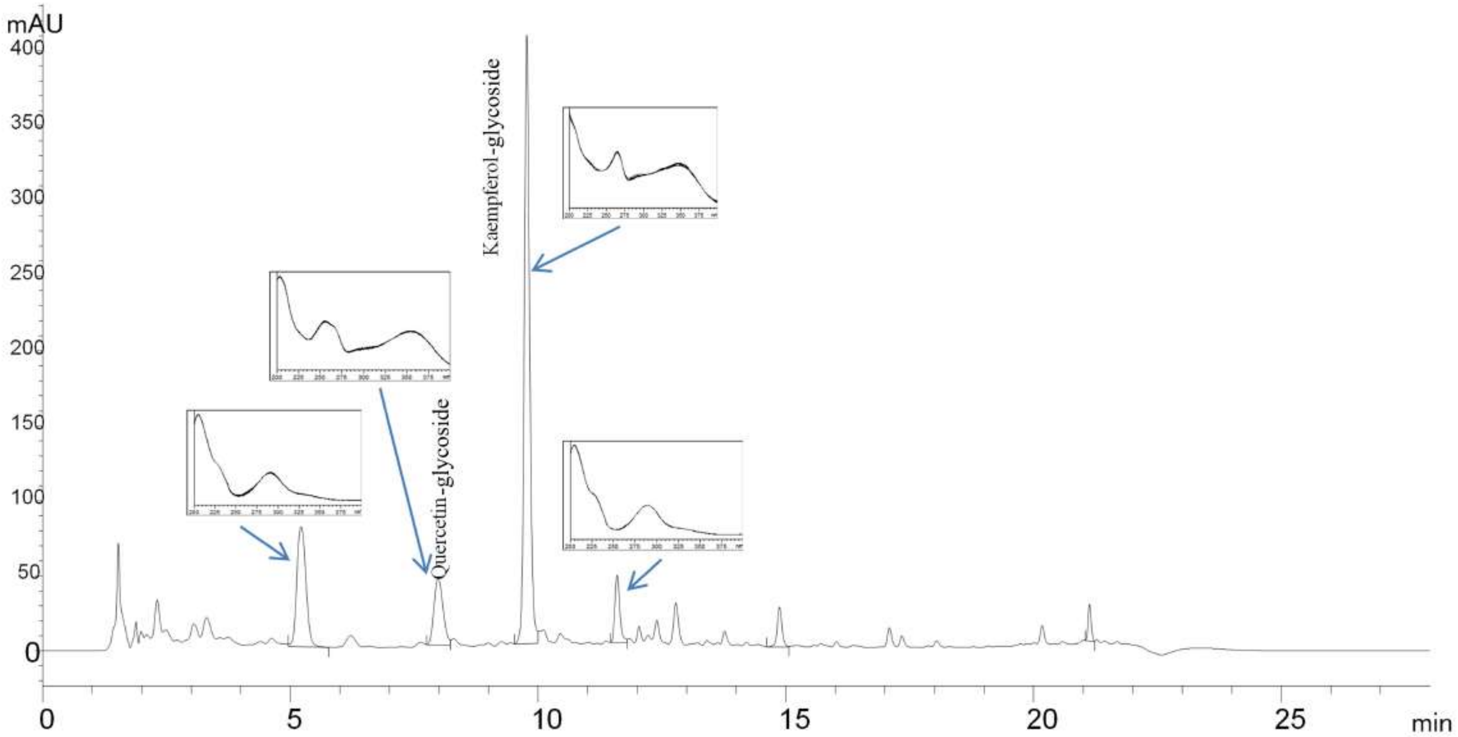

Figure S3: GC-MS chromatogram of *P × euramericana*.
